# Strong Evidence for a Genetic Contribution to Late-Onset Alzheimer’s Disease Mortality: A Population-Based Study

**DOI:** 10.1371/journal.pone.0077087

**Published:** 2013-10-08

**Authors:** John S. K. Kauwe, Perry G. Ridge, Norman L. Foster, Lisa A. Cannon-Albright

**Affiliations:** 1 Department of Biology, Brigham Young University, Provo, Utah, United States of America; 2 Center for Alzheimer’s Care, Imaging and Research, Department of Neurology, University of Utah, Salt Lake City, Utah, United States of America; 3 Genetic Epidemiology, Department of Medicine, University of Utah School of Medicine, Salt Lake City, Utah, United States of America; 4 George E. Wahlen Department of Veterans Affairs Medical Center, Salt Lake City, Utah, United States of America; Pasteur Institute of Lille, France

## Abstract

**Background:**

Alzheimer’s disease (AD) is an international health concern that has a devastating effect on patients and families. While several genetic risk factors for AD have been identified much of the genetic variance in AD remains unexplained. There are limited published assessments of the familiality of Alzheimer’s disease. Here we present the largest genealogy-based analysis of AD to date.

**Methods:**

We assessed the familiality of AD in The Utah Population Database (UPDB), a population-based resource linking electronic health data repositories for the state with the computerized genealogy of the Utah settlers and their descendants. We searched UPDB for significant familial clustering of AD to evaluate the genetic contribution to disease. We compared the Genealogical Index of Familiality (GIF) between AD individuals and randomly selected controls and estimated the Relative Risk (RR) for a range of family relationships. Finally, we identified pedigrees with a significant excess of AD deaths.

**Results:**

The GIF analysis showed that pairs of individuals dying from AD were significantly more related than expected. This excess of relatedness was observed for both close and distant relationships. RRs for death from AD among relatives of individuals dying from AD were significantly increased for both close and more distant relatives. Multiple pedigrees had a significant excess of AD deaths.

**Conclusions:**

These data strongly support a genetic contribution to the observed clustering of individuals dying from AD. This report is the first large population-based assessment of the familiality of AD mortality and provides the only reported estimates of relative risk of AD mortality in extended relatives to date. The high-risk pedigrees identified show a true excess of AD mortality (not just multiple cases) and are greater in depth and width than published AD pedigrees. The presence of these high-risk pedigrees strongly supports the possibility of rare predisposition variants not yet identified.

## Introduction

Alzheimer’s disease (AD) is the most common dementing disease and a pressing international healthcare concern affecting 24 to 35 million people worldwide. With the rapidly aging population, AD incidence is predicted to increase from 1 in 200 to 333 people now [1–3] to 1 in 85 people by 2050 [2]. A better understanding of genetic factors in AD would aid diagnosis and provide new treatment approaches. Genetics clearly plays a role in AD. Early-onset AD, or AD beginning before the age of 65, can be caused by one of more than 200 sequence variants in the amyloid beta precursor protein (APP), presenilin 1 (PSEN1), or presenilin 2 (PSEN2) genes [4–6]. However, just 1% of AD cases have a young age-of-onset and consequently late-onset AD (LOAD) warrants the most attention.

LOAD is also strongly influenced by genetics, but a clear Mendelian pattern of inheritance is often not observed. There are several factors that could account for this even if causative mutations for LOAD exist. 1) The chance for censoring bias increases with increasing age as family members die from other causes before the disease is manifest. 2) A definitive evaluation and autopsy are less likely to be performed in late- as compared to early-onset cases. 3) Younger individuals with AD are more likely to participate in research, particularly clinical drug trials. And lastly, family members are less likely to attribute cognitive changes to disease rather than an illness incident to aging, perhaps because of continuing societal stigma. Although older individuals occasionally have a genetic variant that typically causes early-onset AD [7], no genetic variants that cause LOAD have been discovered. Because of the difficulties in identifying familial LOAD, most attention has been focused on discovering genetic risk factors. However, despite considerable effort, genetic factors leading to LOAD have been difficult to fully explain. Thus far, genetic variants associated with LOAD only convey an added risk of disease. The most important genetic factor in LOAD is the ε4 allele of apolipoprotein E (APOE) [8]. Inheriting one ε4 allele increases risk for AD 4-fold while inheriting two ε4 alleles increases risk 13-fold. Genome-wide association studies (GWAS) are one strategy to identify genetic risk factors. GWAS studies have provided evidence that several gene variants in addition to APOE are associated with LOAD [9–12]. Unfortunately, these variants have only a very modest effect on risk and all GWAS-identified variants contribute only 20-30% of the genetic risk for LOAD [9]. Furthermore, data needed to power GWAS studies rely on large sample sizes of unrelated individuals and have insufficient statistical power to detect rare genetic variants, even if their effect in an individual is substantial or even causative. There is evidence that rare genetic variants play an important role in the risk of LOAD. Whole genome and exome sequencing studies have identified rare variants with large effects on LOAD risk [13,14]. Additional strategies sensitive to rare genetic variants are needed to more rapidly advance our understanding of the genetics of LOAD.

Family-based studies provide a promising approach to identify rare variants cause LOAD or result in a substantial risk. Population-based studies have contributed greatly to our understanding of AD; types of study design include linkage analysis [4,15,16], twin studies [17,18], initial AD population risk studies [19], and environmental factors [20,21]. Family-based studies have suggested a significant genetic role in LOAD by finding that first degree relatives of patients with AD have a two- to four-fold increased risk of dementia from ages 65-80 [22], A weakness of such studies though is that probands were referred for diagnostic evaluation and participation in research studies and such cases invariably report a strong family history of dementia. Furthermore, the disease status of relatives depends upon informant report rather than medical records. The Utah Population database (UPDB) offers a unique resource with the opportunity to combine the advantageous attributes of both population-based and family-based studies. Case ascertainment does not rely on individuals presenting clinically, but rather medical records can be used to identify disease. It is possible to identify multi-generational relatedness and genealogical records are as complete as possible and often verified by several family members as documented in church records, thus avoiding some of the major pitfalls of informant report alone. Here we present the largest genealogical-based analysis of LOAD to date. UPDB death certificates that attributed death at any level to AD were used to test the hypothesis of a genetic contribution to LOAD.

## Methods

### Ethics Statement

All work has been conducted under approval from the Institutional Review Boards at Brigham Young University and the University of Utah as well as specific approvals required by The Utah Population Database.

### The Utah Population Database

The UPDB is a population based resource linking electronic health data repositories for the state, including death certificates, with the computerized genealogy of the Utah pioneers, early Northern European settlers in Utah from the mid 1800s, and their modern day descendants. The original Utah genealogy data in the UPDB was provided by the Family History Library of the Church of Jesus Christ of the Latter Day, Saints (LDS or Mormons) in the early 1970s in the form of 4-generation family history data provided by members. This original genealogy consisted of 1.6 million individuals in pedigrees up to 7 generations deep. More recent data comes from genealogy data represented in Utah State vital records, such as the mother, father, and child on a birth certificate. The UPDB is updated regularly with vital statistics records. The UPDB now includes almost 2.5 million individuals who have at least 3 generations of genealogy data and who are descendants of the original Utah pioneers; there are over 470,000 linked Utah death certificates for these individuals. Over 1.25 million of these individuals have at least 12 of their 14 immediate ancestors in the genealogy and over 275,000 of these have a linked Utah death certificate. We limited analysis to this subset to assure equivalent quantity of genealogy data for cases and controls.

Utah death certificates from 1904 to the present have been coded and record-linked to individuals in the UPDB allowing consideration of all relationships among individuals who have a cause-of-death on their Utah death certificate. Approximately 60-70% of Utah death certificates link to an individual in the UPDB genealogy data. In this study we used the UPDB to search for significant familial clustering as evidence of an important genetic contribution to LOAD. This strategy using the UPDB has led to the discovery and validation of vital genetic roles for many other diseases [23–32].

### Case Classification

AD as a coded cause-of-death first appeared in the International Classification of Disease (ICD) Revision 9, and AD also appears in ICD Revision 10 We only considered individuals to have had AD if their death certificate included AD as a primary or contributing cause-of-death, as shown in Table 1. Others were considered unknown. We recognize that requiring the explicit designation of AD on the death certificate is a conservative approach and that many individuals with AD would not be identified because they were not diagnosed during life or the physician completing the death certificate did not judge AD as leading to death. Since death certificates are well known to underestimate the true prevalence of AD [33–38], we chose to favor specificity over sensitivity – individuals with AD on their death certificates are more likely to truly have had the disease than if we had allowed inclusion of less specific ICD codes such as senility or senile dementia that would more fully encompass individuals with AD. Autopsies were only performed in only a small proportion of individuals so we are unable to comment on diagnostic accuracy. However, it is unlikely that adequate clinical evaluations were performed when death certificates failed to indicate a specific cause of dementia. While appropriate caution must be used [39,40], leveraging the information provided by death certificates is an accepted method for assessing AD prevalence and has contributed in valuable ways to AD research [41–44]. The censoring of some AD cases would not bias results but would more likely affect power. Any censoring of AD cases would be uniform across the resource; rates estimated from the UPDB death certificate population would be conservative but unbiased.

**Table 1:** Number of Alzheimer’s deaths identified in the UPDB by ICD code.

**ICD Revision**	**Code**	**Count**
9	331.0	1,019
10	G30	2,979
**Total**		**3,998**

### Genealogical Index of Familiality (GIF)

The Genealogical Index of Familiality (GIF) analysis allows consideration of familial clustering of a disease among a set of individuals and tests for excess relatedness by comparison with the average relatedness of matched individuals randomly selected from the same population. We calculated the average pairwise relatedness for all possible pairs of all individuals in the UPDB with AD included in their death certificate as either the primary or secondary cause of death (n=3,998). Pairwise relatedness was measured using the Malecot coefficient of kinship [45]. This “case” average relatedness was compared to the expected pairwise relatedness of a group of similar individuals in the UPDB. One thousand matched control sets were chosen for the set of AD death cases; each control set included one sex-, 5-year birth year-, and birthplace- (Utah or not) matched randomly selected UPDB control that had a Utah death certificate. The average relatedness was calculated for each of the 1,000 sets of controls. For evaluation of empirical significance, the average case relatedness was compared to the distribution of the 1,000 average control relatedness measures.

The GIF test for excess relatedness provides evidence for excess relatedness of cases over that expected in the UPDB population; however, the excess relatedness could be due to either shared genes, shared environment, or a combination of both. To evaluate the genetic component of relatedness the contribution to the GIF statistic can be parsed to allow consideration by pairwise genetic distance (or relationship), and considered in graphical form. Pairwise genetic distance = 1 represents parent/offspring; 2 represents siblings or grandparent/grandchild; 3 represents, for example, avunculars; 4 represents, for example, first cousins, and so forth. In order to discriminate excess relatedness that could be due to a genetic contribution, we perform the Distant GIF (dGIF) test. This test is performed in the same way as the GIF test, but all relationships closer than first cousins are ignored; thus allowing for a test of significant excess relatedness when only distant relationships are considered, thus minimizing the effects of shared environment.

### Relative Risks (RR) in Relatives

Relative risks (RR) in relatives is another method that is commonly used to provide evidence for a genetic contribution to a phenotype of interest. For this analysis, RRs were estimated as the ratio of the disease rate among relatives of affected probands to the disease rate in the population of individuals in the UPDB with a Utah death certificate. To estimate RRs for a cause-of-death in the UPDB we estimate the rate of death for the cause (e.g. AD) by cohort. To estimate the rates for AD death, all 277,141 individuals in the UPDB with a death certificate were assigned to one of 133 cohorts by sex, birth year (5 year ranges), and birthplace (Utah or not); this provided the denominator for AD death rate estimation. The numerator for each cohort is the number of individuals with a death certificate indicating AD as a cause-of-death. To estimate the RR for first-degree relatives, for example, we identify all first-degree relatives of all AD deaths who have a death certificate, by cohort (without duplication); we calculate the expected number of AD deaths among these relatives by applying the cohort-specific death rate to the number of deceased relatives in each cohort and then summing over all cohorts. We estimate RR as the ratio of the observed number of AD deaths among the relatives to the expected number of AD deaths calculated in the UPDB identified subjects. Confidence intervals for the RR are calculated as shown in Agresti [46]. Significantly elevated RRs in close relatives may represent either shared genetics or shared environment; however, significantly elevated RRs in distant relatives, who are unlikely to share lifestyle, are indicative of a genetic contribution to risk.

### Identification of High Risk Pedigrees

Pedigrees with a statistically significant excess of deaths from AD can be identified in the UPDB. All descendants of a set of UPDB founders (individuals without parents in UPDB) who have a death certificate are considered as a pedigree. The expected number of AD deaths among the deceased descendants is calculated by applying the cohort-specific AD death rates to the numbers of deceased descendants in each cohort. The ratio of observed to expected AD deaths among deceased descendants is used to test the hypothesis of a significant excess of AD-related deaths. The use of internally estimated death rates controlled for sources of bias that may influence death certificate information.

## Results

Almost 4,000 individuals in the UPDB with at least 3 generations of genealogy data and at least 12 of their 14 immediate ancestors included in the UPDB have a cause-of-death of AD on their death certificate. The number of individuals by ICD 9 revision and code is shown in Table 1. No deaths from AD before the age of 35 years were observed. Table S1 shows the age-at-death distribution for the 3,998 individuals with an AD cause-of-death who are analyzed here; for 2,244 of these AD deaths, AD was the primary cause-of-death.

Summary results for the GIF test for excess relatedness are: number of individuals considered (3998), the case GIF (5.54), the mean control GIF (4.83), the p-value for the overall GIF (<0.001), the case dGIF (4.14), the mean control dGIF (4.39), and the p-value for the distant GIF test (0.005). When all individuals dying from AD are considered as a group, there is overall significant evidence for excess relatedness (GIF p < 0.001); in addition, when relationships closer than first cousins are ignored, a significant excess relatedness is still observed (dGIF p = 0.005), strongly supporting a genetic contribution to this phenotype. Few deaths occurring before age 65 years in the UPDB resource were attributed to AD on a Utah death certificate (n = 62); this sample size is too small to be informative with the GIF analysis.

**Figure 1:**
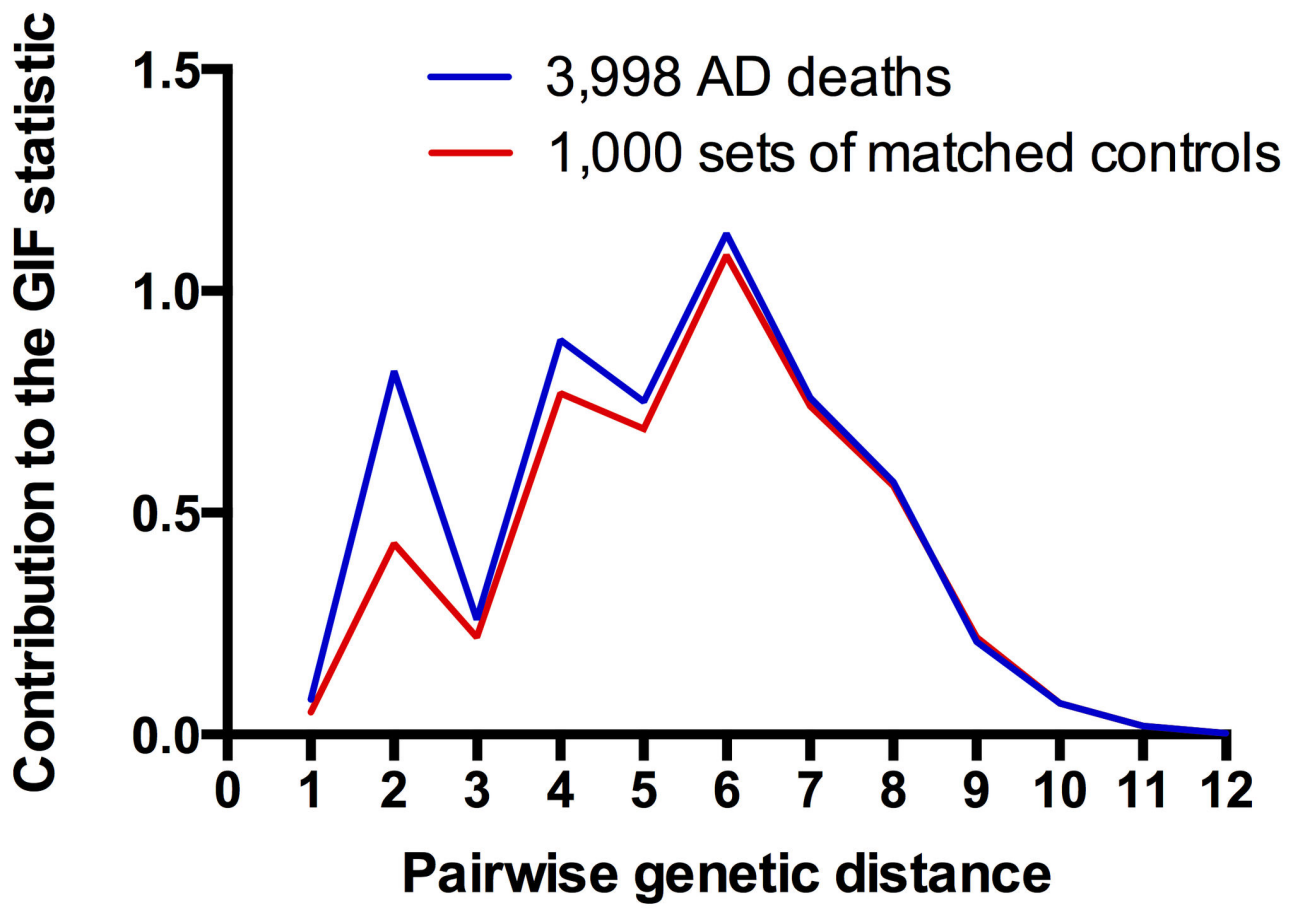
Contribution to the GIF statistic for Alzheimer’s disease mortality relatedness by pairwise genetic distance for cases and controls.

Figure 1 shows the contribution to the overall GIF statistic by pairwise genetic distance for cases and for controls. It is clear in this figure that relatedness of individuals dying from AD is higher than expected for both close and distant relationships; this excess extends out to a pairwise genetic distance of at least 8 (equivalent to third cousins).

Relative Risk estimates for death from AD among first-, second-, and third-degree relatives of individuals dying from AD are shown in Table 2. This table displays the total number of relatives with a death certificate, the number of relatives with a death certificate indicating AD contributed to death (observed), the expected number of AD deaths (expected) among these relatives, the significance value for the test (p-value), the RR estimate, and 95% confidence interval (95% CI) for the RR. RRs for death from AD among first-, second-, and third-degree relatives of individuals dying from AD were all significantly elevated.

**Table 2:** Estimated Relative Risks for death from Alzheimer’s disease among first, second and third degree relatives of individuals dying from Alzheimer’s disease, separated by those dying before age 65, and those dying at all ages.

**Age at Death**	**Relative**	**# relatives**	**observed**	**expected**	**p-value**	**Relative Risks in Relatives**	**95% CI**
All	First-degree	16,909	475	275.1	<0.000001	1.73	1.57-1.89
	Second-degree	28,656	283	230.0	5.2e-4	1.23	1.09-1.38
	Third-degree	78,881	1,400	1,296.4	0.0041	1.08	1.02-1.14
Under 65	First-degree	132	4	2.3	0.196	1.76	0.48-4.50
	Second-degree	499	15	7.0	0.006	2.13	1.19-3.52
	Third-degree	1,124	20	13.2	0.072	1.51	0.92-2.34

We also considered the RR for death from AD among the first-, second-, and third-degree relatives of individuals dying from AD before age 65 years (Table 2). Sample sizes are much smaller due to the small number of young AD deaths in the UPDB, leading to large confidence intervals for RRs. All RRs were elevated, but only the second-degree RR was significantly elevated (RR = 2.13; 95% CI 1.19, 3.52; p = 0.006).

**Figure 2:**
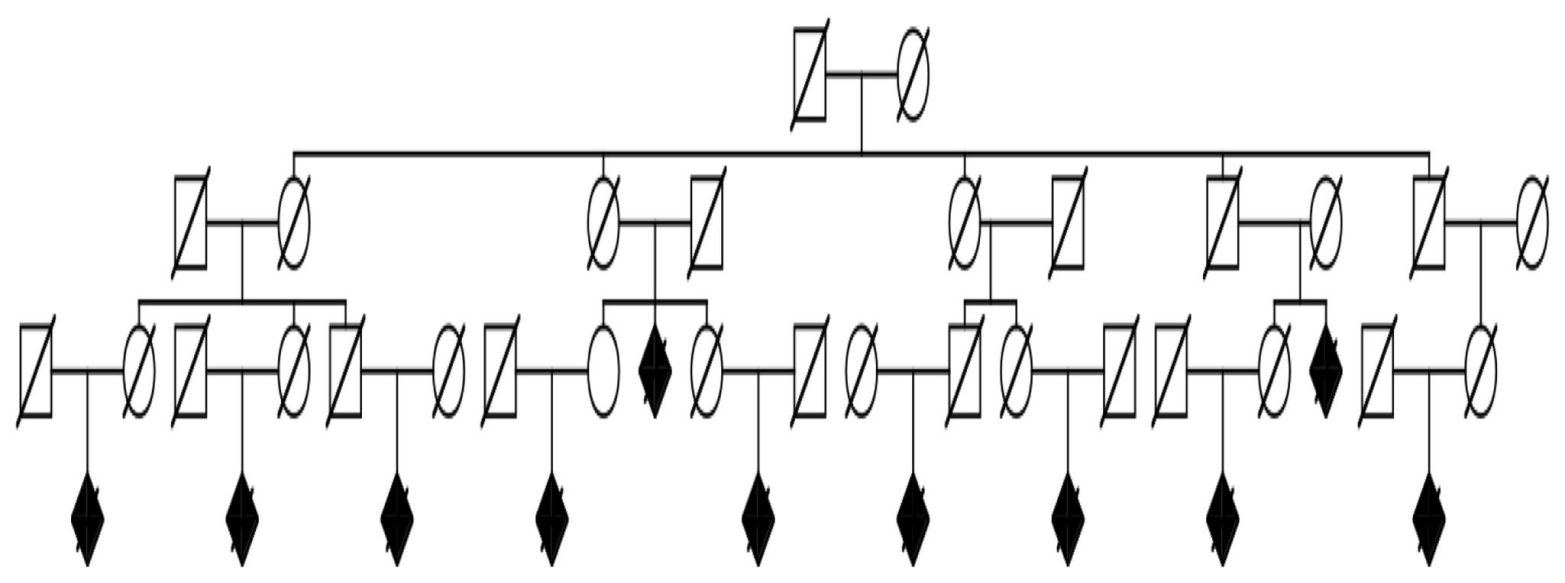
Example of a high-risk Alzheimer’s disease mortality pedigree. Squares are males, circles are females, and diamonds are gender-censored (unspecified gender) individuals. Individuals with a diagonal line are deceased, and shaded shapes are Alzheimer’s disease cases; unaffected siblings are not shown. The founder has more than 4,100 descendants; 11 died from Alzheimer’s disease, only 3.2 Alzheimer’s disease deaths were expected (p= 0.0031).

Figure 2 shows an example high-risk Utah AD pedigree identified in the UPDB. The founding couple had over 4,100 descendants with data in the UPDB. Eleven of these descendants died from AD while only 3.2 AD deaths were expected. There were more than 60 additional high-risk AD death pedigrees (p<0.01) with at least 5 individuals dying from AD identified in the UPDB. As might be expected from the narrow window of view to AD deaths in Utah (only ICD9 and ICD10 coded death certificates), most individuals dying from AD are in the same generation (few parent offspring pairs). This is also evident in Figure 1 where dips associated with a smaller number of pairs of genetic distances 1, 3 and 5, which represent individuals from different generations.

## Discussion

Our understanding of the genetic basis for Alzheimer’s disease has dramatically increased in the past few years. Nevertheless, a significant proportion of genetic variance in risk for disease and the majority of high-risk late-onset AD pedigrees remain unexplained. Using a unique population-based resource in the state of Utah that combines genealogy data from the mid 1800s with death certificates from the early 1900s, we have presented an unbiased view of the familial clustering of Alzheimer’s disease mortality as well as strong evidence for a genetic contribution for late-onset AD. The methods used in this study have previously provided evidence for a heritable component to many diseases, including: influenza mortality, rotator cuff disease, asthma mortality, pancreatic cancer, and lumbar disc disease, among others [23–28]. Studies of Utah high-risk pedigrees identified in the UPDB have lead to the discovery of multiple disease predisposition genes including *BRCA1, BRCA2, p16*, and *HPC2/ELAC2*, among others [29–32]. The UPDB data analyzed represents a homogeneous population that has been shown to be genetically representative of Northern Europe, with normal levels of inbreeding levels and thus not representative of a genetically isolated group [47,48].

The vast majority of AD deaths in Utah occurred late in life, although we cannot determine age at symptom onset in the database. Comparison of the RR results from the potential early-onset AD cases (represented by early AD deaths, Table 2) with the overall RR results shows that the early-onset AD cases had very little impact in the overall results presented. The various analyses presented strongly support a genetic contribution to the observed clustering of individuals dying from AD. The GIF analysis showed that pairs of individuals dying from AD were significantly more related than expected for a similar group of individuals randomly selected from Utah deaths. An excess of relatedness was observed for both close, and distant relationships. RRs for death from AD among relatives of individuals dying from AD were significantly elevated, for both close and more distant relatives.

Multiple pedigrees showing a significant excess of death from AD have been identified in the UPDB. These pedigrees differ from published high-risk AD pedigrees in that the phenotype considered is “AD that contributed to death”. They also differ in their typically very large generational depth and breadth of identified individuals, which are both considerably larger than published AD pedigrees. In addition, because they have been ascertained from a population and we have considered population rates of AD death in this population, the pedigrees are truly high-risk (with a significant excess of observed over expected deaths) rather than just pedigrees with a large number of related AD deaths. As such, these pedigrees are ideal for future genetic studies of AD.

This study used a uniform, consistent source for all diagnoses, namely AD that contributed to cause-of-death as confirmed by presence on a death certificate. Thus the results are not limited by the bias caused from selected ascertainment of cases or requiring informant recall for diagnoses. The most significant limitation of this analysis is the narrow window of view to identify individuals diagnosed with death from AD. This results from the relatively short period of time for which coding for this diagnosis has existed (only present in ICD 9 and 10). These effects limit our ability to identify cases that might be related across different generations (e.g. grandparent/ grandchild or avunculars), but not our ability to identify related cases who died in the same cohort (e.g. distant cousins). Although individuals dying from AD may have been censored from our observation in this resource, the assumption can be made that cases are uniformly censored across the data set, leading to conservative, but unbiased, estimates of familiality due to potential inclusion of censored cases in estimation of death rates and selection of controls. An additional limitation is the recognized under-reporting of AD as a cause-of-death on death certificates, which could have resulted in lower estimates of AD mortality rates overall. On the other hand, the physicians’ willingness to specify this cause-of-death rather than using a general term such as senility or senile dementia, or omitting its mention altogether likely indicates that an evaluation was performed when cause was considered. Although in the ideal world disease classification would be based upon autopsy findings or review of medical records and consensus diagnosis, this is impractical in a large population-based study and the direct medical information the treating physician provides in the death certificate is a reasonable alternative. Because we apply the internal AD death rates to the UPDB population of individuals with death certificates uniformly, we assume there is no resulting bias in risk estimates.

The presence of familiality in AD is unsurprising. ApoE genotype does affect risk of AD among family members, but not to the degree observed in this study. In one report, among relatives in the ApoE 3/3 group, the lifetime risk for AD by age 90 was greater than 3 times the expected proportion of ε4 carriers [49]. In addition, by age 93 at least 50% of ε4/ε4 individuals did not develop dementia. Although we have not determined apoE status in family members, we believe it is unlikely that inheritance of a particular apoE genotype explains our observations. Likewise, variants that confer only a modest increased risk of AD are unlikely to be explanatory. Because older individuals are at higher risk for AD, there may be some confounding of longevity heritability with AD heritability. Such confounding does not invalidate the results, but rather indicates the complexity of the study of the genetics of all late-onset complex disease.

This study of AD heritability does not allow determination of the mechanisms that lead to genetic predisposition to death from AD. This will require in depth assessment of families with high familiality for AD and comparison with families in which the prevalence of AD diagnoses are either typical or under-represented. We have identified multiple pedigrees with a significant excess of late-onset AD deaths. The presence of these high-risk pedigrees strongly supports the possibility of rare predisposition variants not yet identified. We propose the study of these pedigrees to identify the missing heritability (rare variants) predisposing to AD, as well as to better understand mechanisms and potential environmental factors.

## Supporting Information

Table S1 **Summary of age at death for the 3,998 individuals with an Alzheimer’s cause of death.**
(DOCX)
