# Supplementary material for: Strong Evidence for a Genetic Contribution to Late-Onset Alzheimer’s Disease Mortality: A Population-Based Study
Source: PLoS One. 2013 Oct 8;8(10):e77087. doi: 10.1371/journal.pone.0077087 (PMC3792903; doi:10.1371/journal.pone.0077087)
Supplement: Table S1 — Summary of age at death for the 3,998 individuals with an Alzheimer’s cause of death. (DOCX) [file pone.0077087.s001.docx]

**Table S1. Summary of age at death for the 3,998 individuals with an Alzheimer’s cause of death.**

**Age Number of deaths**

35 - 39 1

40 - 44 0

45 - 49 0

50 - 54 4

55 - 59 19

60 - 64 38

65 - 69 101

70 - 74 219

75 - 79 561

80 - 84 982

85 - 89 1093

90 - 94 726

95 - 99 225

100 - 104 27

105 - 109 2

total 3998
